# Pain perception in acute model mice of Parkinson’s disease induced by 1-methyl-4-phenyl-1,2,3,6-tetrahydropyridine (MPTP)

**DOI:** 10.1186/s12990-015-0026-1

**Published:** 2015-05-17

**Authors:** Jihye Park, Chae-Seok Lim, Hyunhyo Seo, Chung-Ah Park, Min Zhuo, Bong-Kiun Kaang, Kyungmin Lee

**Affiliations:** Neurobiology Laboratory, College of Natural Sciences, Seoul National University, 599 Gwanangno, Seoul, 151-747 South Korea; Behavioral Neural Circuitry and Physiology Laboratory, Department of Anatomy, Brain Science & Engineering Institute, Kyungpook National University Graduate School of Medicine, 2-101, Dongin-dong, Jung-gu, Daegu, 700-842 South Korea; Department of Physiology, Faculty of Medicine, University of Toronto, The center for the study of pain, 1 King’s College Circle, Toronto, ON M5S 1A8 Canada

**Keywords:** Dopaminergic pathway, Subthalamic nucleus, Inflammation, Astrogliosis

## Abstract

**Background:**

Pain is the most prominent non-motor symptom observed in patients with Parkinson’s disease (PD). However, the mechanisms underlying the generation of pain in PD have not been well studied. We used a 1-methyl-4-phenyl-1,2,3,6-tetrahydropyridine (MPTP)-induced mouse model of PD to analyze the relationship between pain sensory abnormalities and the degeneration of dopaminergic neurons.

**Results:**

The latency to fall off the rotarod and the total distance traveled in round chamber were significantly reduced in MPTP-induced PD mice, consistent with motor dysfunction. MPTP-treated mice also showed remarkably shorter nociceptive response latencies compared to saline-treated mice and the subcutaneous injection of L-3,4-dihydroxyphenylalanine (L-DOPA) partially reversed pain hypersensitivity induced by MPTP treatment. We found that degeneration of cell bodies and fibers in the substantia nigra pars compacta and the striatum of MPTP-treated mice. In addition, astrocytic and microglial activation was seen in the subthalamic nucleus and neuronal activity was significantly increased in the striatum and globus pallidus. However, we did not observe any changes in neurons, astrocytes, and microglia of both the dorsal and ventral horns in the spinal cord after MPTP treatment.

**Conclusions:**

These results suggest that the dopaminergic nigrostriatal pathway may have a role in inhibiting noxious stimuli, and that abnormal inflammatory responses and neural activity in basal ganglia is correlated to pain processing in PD induced by MPTP treatment.

## Background

Parkinson’s disease (PD) is one of the best examples of a neurodegenerative disease which manifests comorbid pain. About 40-60 % of patients with PD experience various types of acute or chronic pain [1]. To date, these types of pain have been well characterized in human PD patients [2], although the exact mechanism of pain perception in PD is still elusive. In addition, there are no apparent remedies to cure PD-induced pain in the field of clinical neurology. For this reason, it is necessary to define the pathophysiology in rodent models of PD.

1-methyl-4-phenyl-1,2,3,6-tetrahydropyridine (MPTP) is a neurotoxin discovered in 1982 and has been widely used to establish an animal model of PD. We chose MPTP among the various dopaminergic neurotoxins including 6-hydroxydopamine (6-OHDA) and rotenone to generate a PD model since this compound has specific advantages: 1) MPTP is known as the only neurotoxin which causes degeneration of dopaminergic neurons in humans diagnosed with PD, 2) the administration of MPTP does not require technical skills or a particular apparatus, and 3) MPTP reliably induces degeneration of cell bodies and fibers in the substantia nigra pars compacta (SNpc) and in the striatum [3].

Dopamine receptors (D_1_ and D_2_) in the striatum are involved in nociception [4], and therefore there have been many studies to assess the alterations of nociceptive sensitivity in rodent PD models [5, 6]. However, none of these revealed how MPTP affects the sensitivity, with or without pain induction, of mechanical and thermal nociception in mice. In this study, we found that the alterations of mechanical and thermal nociceptive thresholds due to depletion of dopaminergic neurons and fibers in MPTP-induced PD model mice. We also investigated that an acute MPTP-induced PD mouse is an appropriate model to study the mechanism of pain symptoms in PD.

Although many drugs have been used for treatment of PD patients, L-DOPA has thus far proven to be the most effective medication to alleviate motor dysfunction in PD and antinociceptive effects of L-DOPA has been also investigated [7]. However, it has been well known that long-term medication of L-DOPA is highly accompanied by several complications such as disabling fluctuations and dyskinesias [8] overwhelming its therapeutic benefits.

High frequency deep brain stimulation (DBS) of subthalamic nucleus (STN) (STN-DBS) has been considered as a powerful therapeutic tool to treat severe motor symptoms of PD [9] by controlling neural circuitry forming functional loop within the basal ganglia including striatum, globus pallidu (GP), and STN. Recently, several clinical studies have demonstrated that STN-DBS could be effective on pain perception in PD patients [10, 11] although the mechanism underlying the therapeutic effect of STN-DBS on pain modulation remains unraveled yet. In this study, we hypothesized that dysfunction of neural circuitry via inflammatory responses and abnormal neural activity within basal ganglia could be implicated in pain processing. Thus, the aim of this study was to explore and compare the inflammatory changes and neural activity in basal ganglia of PD animal model induced by MPTP treatment.

## Results

### Acute administration of MPTP induces depletion of dopaminergic neurons in mice

**Fig. 1:**
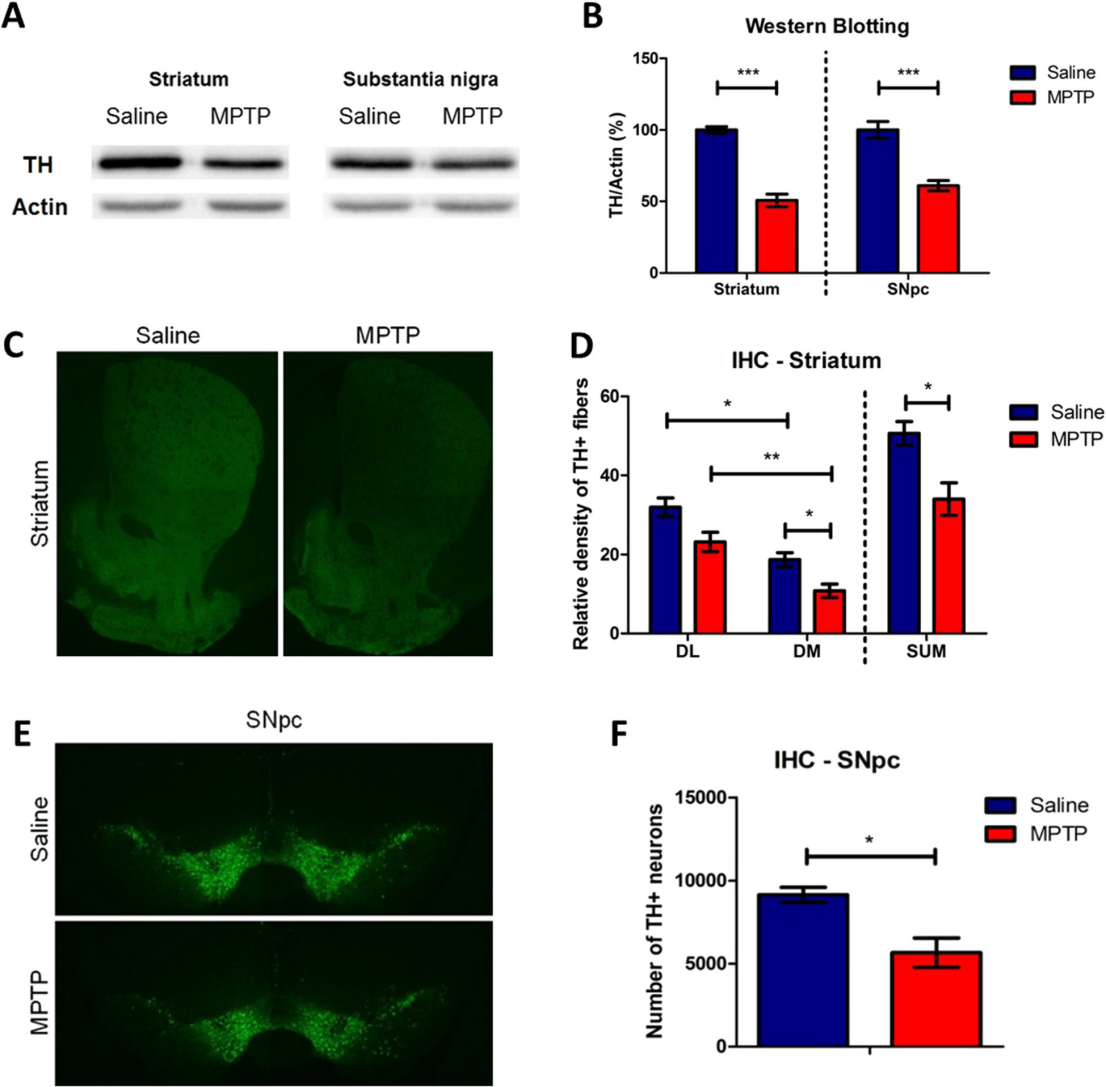
Depletion of dopaminergic neurons both in the striatum and substantia nigra (SN) induced by MPTP injection in mice. **a** A representative western blot image using a tyrosine hydroxylase (TH) antibody. **b** Quantification of normalized TH expression levels in the striatum and the substantia nigra pars compacta (SNpc) (*n* = 5 per group, *p* < 0.0001, striatum; *p* < 0.0005, SNpc, unpaired Student’s *t*-test). **c** Visualization of loss of dopaminergic neurons by TH immunostaining in the striatum. **d** Quantification of reduction of striatal fibers in MPTP-treated mice assessed by TH immunostained optical density (*n* = 3 per group, *p* < 0.05, dorsomedial (DM) or dorsal striatum (SUM), unpaired Student’s *t*-test; *p* < 0.05, dorsolateral versus dorsomedial striatum in saline-treated mice, paired Student’s *t*-test; *p* < 0.005, dorsolateral versus dorsomedial striatum in MPTP-treated mice, paired Student’s *t*-test). **e** Visualization of loss of dopaminergic neurons by TH staining in the SNpc. **f** Quantification of reduction of SNpc dopaminergic neurons in MPTP-treated mice (*n* = 3 per group, *p* < 0.05, unpaired Student’s *t*-test). DM, Dorsomedial striatum; DL, Dorsolateral striatum

In the current experiment, an acute MPTP administration protocol [3] was applied to generate Parkinson’s disease (PD) model mice. We first confirmed this PD model by western blot and immunohistochemistry. The protein level of tyrosine hydroxylase (TH) was reduced in the striatum and the substantia nigra pars compacta (SNpc) of MPTP-treated animals compared to saline-treated animals (Fig. 1a, b). Acute MPTP administration also caused the dramatic degeneration of nerve fibers in the striatum (Fig. 1c, d) and dopaminergic neurons in the SNpc (Fig. 1e, f). There was substantial difference in the fluorescent intensity of TH-immunoreactive fibers in the dorsal striatum between saline- and MPTP-treated groups and the magnitude of decrease in the TH-immunoreactive intensity was much larger in the dorsomedial (DM) striatum than the dorsolateral (DL) striatum in MPTP-treated group (Fig. 1c, d). The number of TH-immunoreactive neurons in the SNpc was significantly decreased in MPTP-treated group (Fig. 1e, f). Thus, consistent with the previous report [3], acute administration of MPTP significantly reduced dopaminergic neurons in the SNpc and nerve fibers in the striatum in mice.

### MPTP-induced PD model mice exhibit motor deficits

**Fig. 2:**
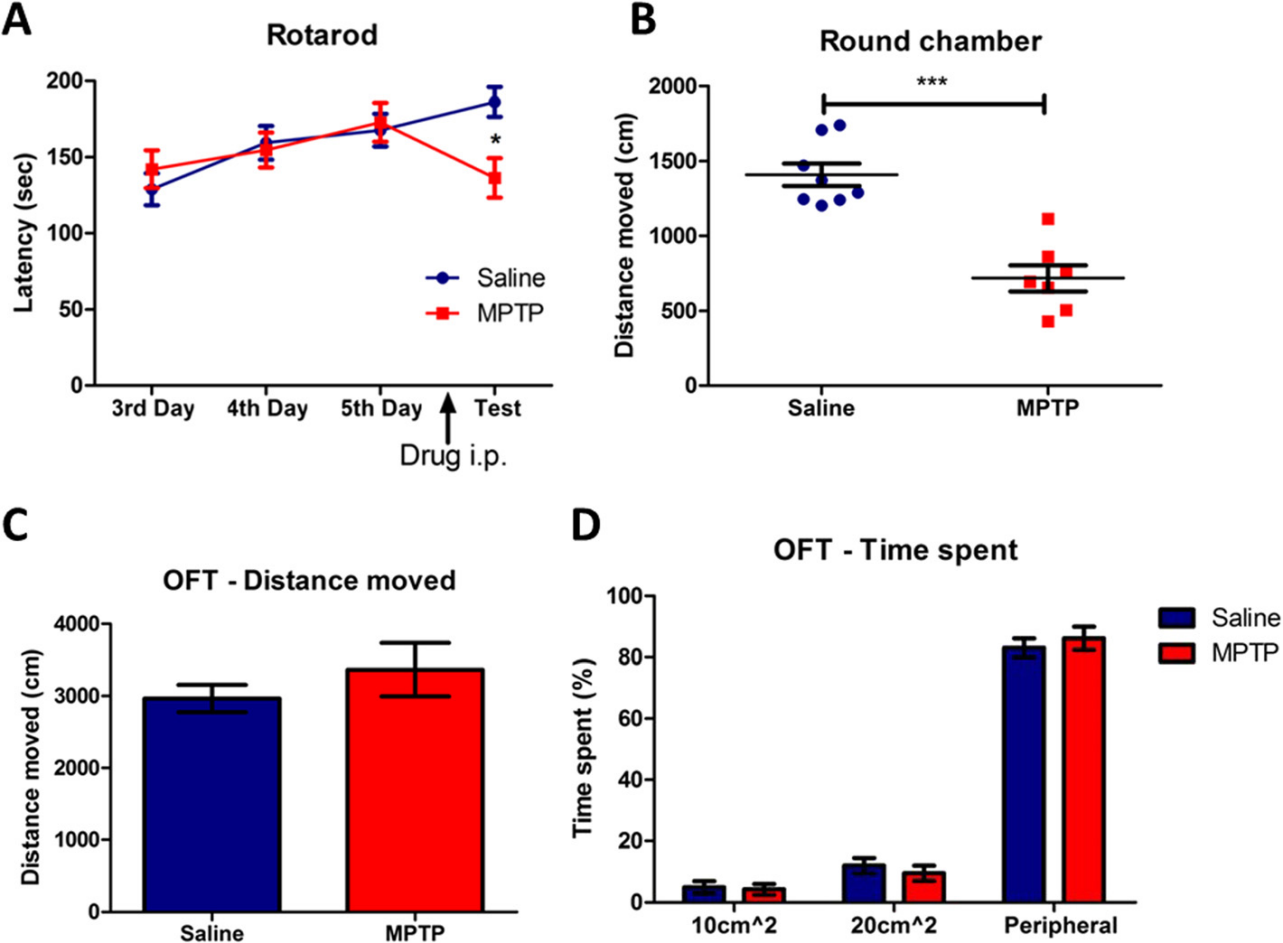
Locomotor activity of MPTP-induced PD model mice. **a** Motor performance of MPTP-treated mice was significantly decreased compared to saline-treated mice in the rotarod test (*n* = 15 per group, interaction *p* < 0.0001; post-hoc *p* < 0.05, two-way ANOVA). **b** A severe locomotor deficit was observed in MPTP-treated mice group (*n* = 8 of saline and *n* = 7 of MPTP group, *p* < 0.0001, unpaired Student’s *t*-test). **c** and (**d**) Saline- and MPTP-treated mice showed no difference in the open field test (OFT) (*n* = 4 per group)

Since the main symptoms of PD are motor deficits [12], we performed the rotarod test, round chamber test, and open field test (OFT), which are commonly used to confirm the phenotypes of PD model animals [13]. Motor performance assessed by rotarod test was significantly decreased after acute MPTP administration (20 mg/kg, 4 times 2 h interval) (Fig. 2a). During the training days, no difference was observed in the rotarod performance between saline- and MPTP-treated mice. Saline-treated mice showed increased rotarod performance on the test day, whereas MPTP-treated mice showed significantly decreased falling latency on the rotarod (Fig. 2a). Moreover, a severe motor deficit in MPTP-treated mice was observed in the round chamber test (Fig. 2b). However, there were no differences in distance traveled or time spent in the center between saline- and MPTP-treated groups in the OFT (Fig. 2c, d), which was consistent with a previous report that acute MPTP administration induced no alteration in mobility in the OFT [14], suggesting no changes in basal anxiety levels of the MPTP-induced PD model mice. Taken together, these results indicate that the acute MPTP administration protocol we used induces motor deficits, a behavioral phenotype of PD model mice.

### Administration of MPTP significantly reduces the basal threshold of nociceptive sensitivity in mice

**Fig. 3:**
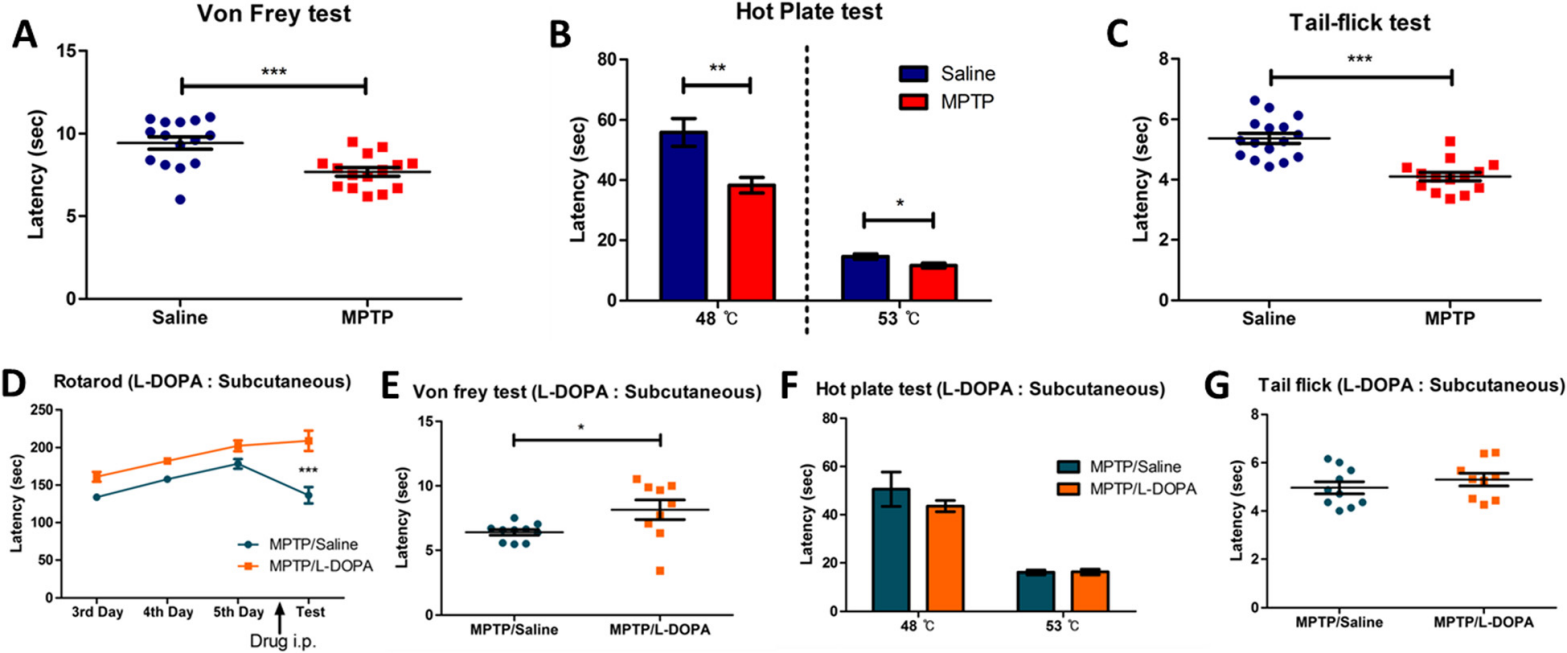
Changes in baseline threshold of nociceptive sensitivity to mechanical and thermal stimulation in MPTP-induced PD model mice and L-DOPA-treated PD model mice. **a** Hindpaw withdrawal latency of MPTP-treated mice was significantly reduced in the von Frey test (*n* = 15 per group, *p* < 0.001, unpaired Student’s *t*-test). **b** The thermal nociceptive response to constant thermal stimulation was increased in MPTP-treated group in the hot plate test (*n* = 15 per group, *p* < 0.005, 48 °C; *p* < 0.05, 53 °C, unpaired Student’s *t*-test). **c** The nociceptive responses to laser heat stimulation of the tail was increased in MPTP-treated mice (*n* = 16 for saline- and *n* = 14 for MPTP-treated group, *p* < 0.0001, unpaired Student’s *t*-test). **d** Motor dysfunction was eliminated in PD model mice by subcutaneous injection of L-DOPA in the rotarod test (*n* = 10 for saline and *n* = 9 for L-DOPA group, *p* < 0.001, two-way ANOVA). **e** In the von Frey test, MPTP-induced hyperalgesia to mechanical stimuli was ameliorated by subcutaneous injection of L-DOPA (n = 10 for saline and *n* = 9 for L-DOPA group, *p* < 0.05, unpaired Student’s *t*-test). **f** and (**g**) Subcutaneous injection of L-DOPA did not ameliorate MPTP-induced thermal hyperalgesia in the hot plate and the tail flick tests (*n* = 10 for saline and *n* = 9 for L-DOPA group)

Next, we used several nociceptive behavioral tests to measure the nociceptive thresholds of mechanical and thermal stimulation in saline- and MPTP-treated mice. The threshold of basal nociceptive response to mechanical stimulation was reduced in the MPTP-treated group compared to control group in the von Frey test (Fig. 3a). Next, the threshold of basal nociceptive response to thermal stimulation on both the 48 °C and 53 °C hot plate was considerably decreased in the MPTP-treated group (Fig. 3b). In addition, the MPTP-treated group also showed significantly reduced tail flick latency compared to control group (Fig. 3c). Taken together, these results suggest that administration of MPTP with acute protocol to mice reduces basal nociceptive thresholds to mechanical and thermal stimuli.

We then performed the restoration experiments with L-DOPA to verify whether nigrostriatal dopaminergic pathway is implicated in pain processing. To examine whether L-DOPA could ameliorate MPTP-induced hyperalgesia, L-DOPA (40 mg/kg) or saline was subcutaneously injected 45 min prior to each experiment. The motor dysfunction in MPTP-treated mice was fully abrogated by L-DOPA administration (Fig. 3d). The L-DOPA-injected group showed also significantly increased response latency in the von Frey test compared to saline-injected PD model group (Fig. 3e). However, no antinociceptive effect of L-DOPA was observed in the hot plate test and the tail flick test (Fig. 3f, g respectively). These results suggest that the effects of MPTP on nociceptive behaviors were partially abrogated in mechanical allodynia by L-DOPA and dopaminergic pathway is related to pain processing, primarily mechanical allodynia in PD.

### MPTP-induced PD model mice show differential inflammatory responses and neuronal activity in the brain and spinal cord

**Fig. 4:**
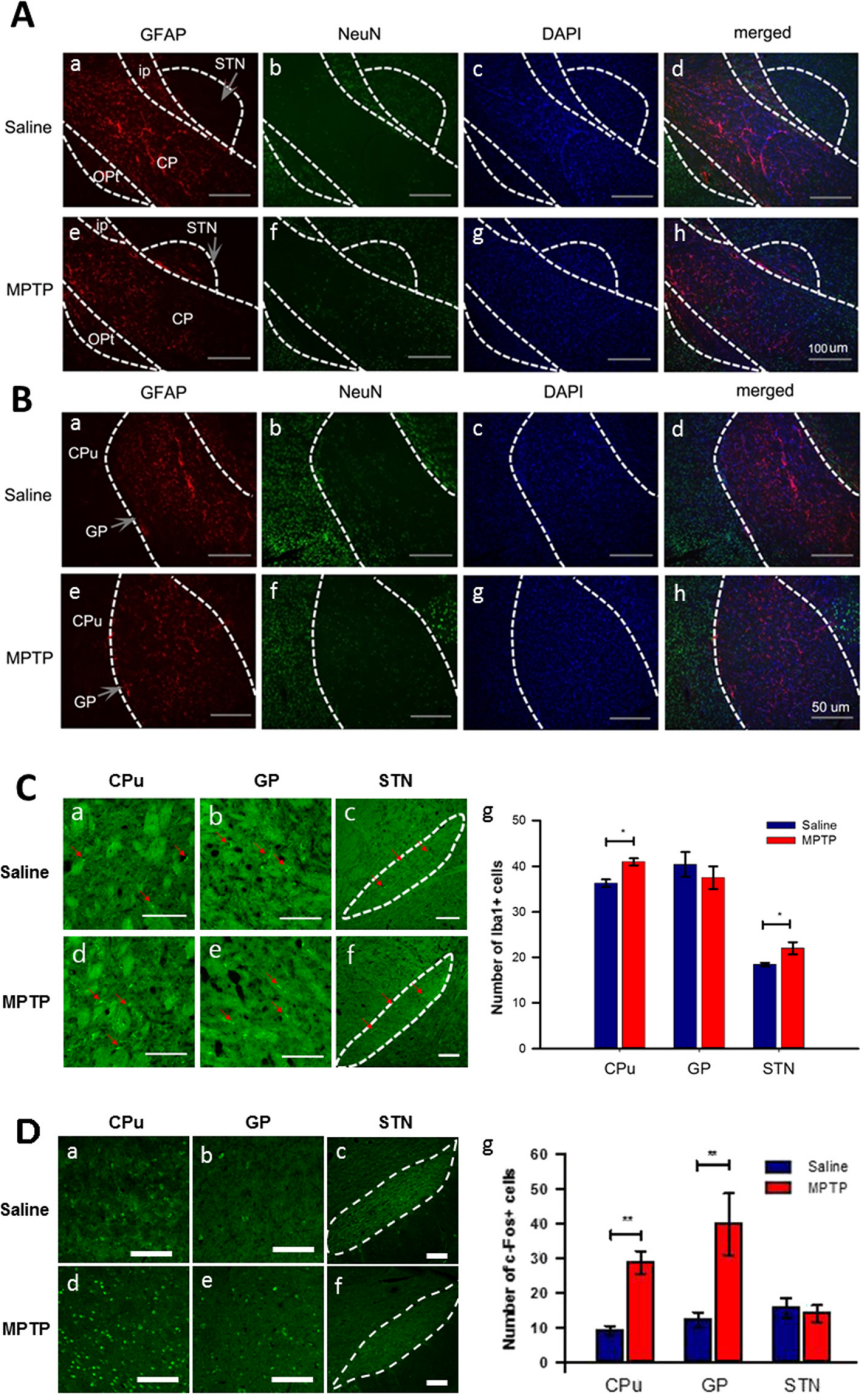
Change of astrocytes and neurons in the basal ganglia of MPTP-administered mice. **a** Change of astrocyte activation and neuron in the STN after MPTP treatment. GFAP-immunoreactivity was increased in the STN after MPTP treatment (Ae-h) compared to saline treatment (Aa-d), whereas there is no difference in NeuN-immunoreactivity between two groups (scale bars = 100 μm) (*n* = 4 per group). **b** Change of astrocyte activation and neuron in the GP after MPTP treatment. There were no differences in GFAP- and NeuN-immunoreactivity in the GP after MPTP treatment (Be-h) compared to saline treatment (Ba-d) (scale bars = 50 μm) (*n* = 4 per group). **c** Change of microglial activity in the CPu, the GP, and the STN after MPTP treatment. The Iba1 expression (red arrows) as a marker for microglial activity was increased in the CPu (Cd) and the STN (Cf), but not in the GP (Ce) after MPTP treatment (Cg) compared with saline treatment (Ca, Cb, and Cc, respectively) (scale bars = 100 μm) (*n* = 5 per group, *p* < 0.001, CPu; *p* < 0.05, STN, unpaired *t*-test). **d** Change of neuronal activity in the caudate-putamen, the GP, and the STN after MPTP treatment. The c-Fos expression as a marker for neuronal activity was remarkably increased in the CPu (Dd) and the GP (De), but not in the STN (Df) after MPTP treatment (Dg) compared with saline treatment group (Da, Db, and Dc, respectively) (scale bars = 100 μm) (*n* = 4 per group, *p* < 0.0001, CPu; *p* < 0.01, GP, unpaired *t*-test). CP, cerebral peduncle; CPu, caudate-putamen; GP, globus pallidus; i.c., internal capsule; Opt, optic tract; STN, subthalamic nucleus

Astrocytes are significantly activated by brain injury or abnormal microenvironment of neural tissues and subsequent deleterious impacts on neurons, which may result in pathological process of the brain, such as neuropathic pain and neurodegenerative diseases [15]. This reactive astrogliosis is involved in molecular and morphological alterations of the astrocytes such as enhancement of GFAP expression and accumulation of α–synuclein [16]. We next searched for astrocyte activation in the basal ganglia subunits such as the globus pallidus (GP) and subthalamic nucleus (STN), which have shown astrogliosis after SNpc lesion by injection of 6-OHDA into the striatum [17]. As shown in other studies [16–18], we found a strong upregulation of GFAP immunoreactivity in the STN after MPTP treatment (Fig. 4a*e*-a*h*) compared to saline treatment (Fig. 4a*a*-a*d*). However, it was difficult to find astrocytic activation in the caudate-putamen (CPu) (data not shown) or GP (Fig. 4b) after acute MPTP treatment, although the astrocytes of the GP showed more numerous processes and a more branched appearance (Fig. 4b*e*-b*h*) compared to those of saline-treated mice (Fig. 4b*a*-b*d*). Many studies on the mechanisms of pain in PD have concentrated on the crosstalk between astrocytes and neurons. Astrogliosis is a main feature of PD to lead neuronal death [19, 20]. To verify the reciprocal regulation of astrocytes to neurons, we checked the neuronal changes using NeuN immunolabeling in the basal ganglia. We found that acute MPTP treatment did not involve the neuronal changes in both STN (Fig. 4a*f*) and GP (Fig. 4b*f*) compared with saline treatment (Fig. 4a*b*, 4b*b*, respectively).

**Fig. 5:**
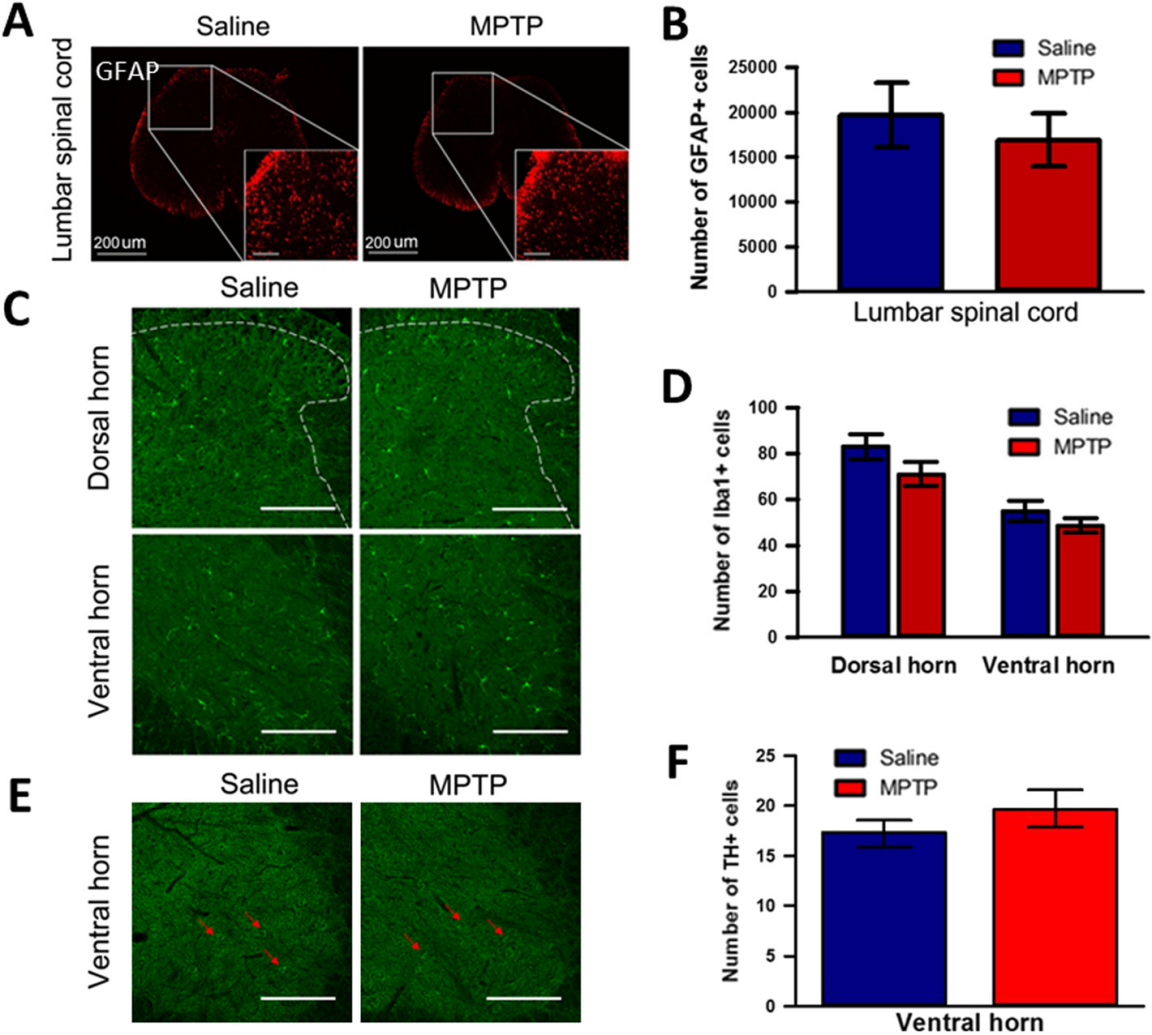
Quantitative analysis of astroglial and microglial activation to MPTP treatment in the spinal cord. **a** Comparison of GFAP-immunopositive astrocytes in the dorsal horn of the lumbar spinal cord between saline- and MPTP-treated mice (scale bars = 200 μm). **b** Saline- and MPTP-treated mice showed no difference in the number of astrocytes in the spinal cord (*n* = 4 per group, unpaired *t*-test). **c** Comparison of Iba1-immunopositive microglia in the dorsal and ventral horn of the lumbar spinal cord between saline- and MPTP-treated mice (scale bars = 200 μm). **d** Saline- and MPTP-treated mice showed no difference in the number of microglial cells in the spinal cord (*n* = 4 per group, unpaired *t*-test). **e** Comparison of TH-immunopositive neurons (red arrows) in the ventral horn of the lumbar spinal cord between saline- and MPTP-treated mice (scale bars = 200 μm). **f** Saline- and MPTP-treated mice showed no difference in the number of TH-positive neurons in the spinal cord (n = 4 per group, unpaired *t*-test)

**Fig. 6:**
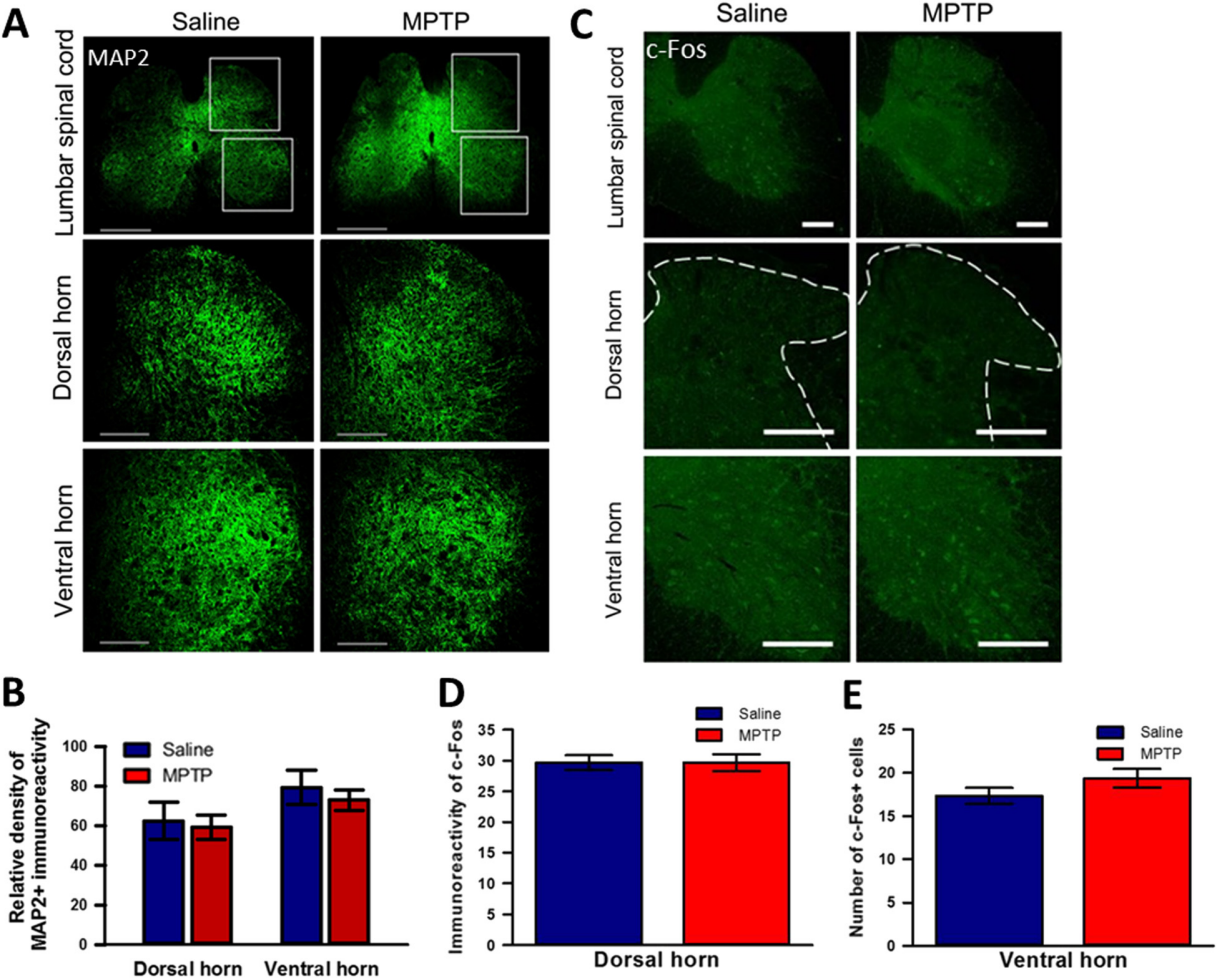
Change of neurons in the spinal cord of MPTP-administered mice. **a** Comparison of MAP2-immunopositive spinal neurons in the dorsal and ventral horn of the lumbosacral spinal cord between saline- and MPTP-treated mice (scale bars = 200 μm). Upper and lower rectangles represent dorsal and ventral horns, respectively. Higher magnification images of these dorsal and ventral horns showed in lower two panels (scale bars = 200 μm). **b** MPTP-treated PD model mice did not exhibit any neuronal changes in either the dorsal or ventral horn of the lumbosacral spinal cord (*n* = 4 per group, unpaired *t*-test). **c** Comparison of c-Fos immunolabelled neurons in the dorsal and ventral horn of lumbar spinal cord between saline- and MPTP-treated mice (scale bars = 200 μm). Higher magnification images of these dorsal and ventral horns showed in lower two panels, respectively (scale bars = 200 m). **d** and (**e**) MPTP-treated PD model mice did not show any differences in neuronal activity in either the dorsal or ventral horn of the lumbar spinal cord (*n* = 4 per group, unpaired *t*-test), respectively

We then examined the microglial activation to confirm the inflammatory responses to MPTP treatment and we also found Iba 1-immunopositive microglial activation in the STN (Fig. 4c*c*, 4c*f*), but not in GP (Fig. 4c*b*, 4c*e*) compared to saline treatment. However, in contrast to GFAP expression, we could observe the increase of microglial activation to show inflammatory responses in the CPu (Fig. 4c*a*, 4c*d*) after acute MPTP treatment. To address whether the dysfunction of SNpc induced by acute MPTP injection affect an interactive circuitry between the basal ganglia subunits, we then investigated the change of neuronal activity in the basal ganglia using c-Fos expression. As shown in the Fig. 4d, neuronal activity was highly increased in the CPu (Fig. 4d*a*, 4d*d*) and the GP (Fig. 4d*b*, 4d*e*) after MPTP treatment. However, MPTP treatment did not cause any increase in the neuronal activity of STN (Fig. 4d*f*) compared to saline treatment. These data suggest that the dynamic networks and neuronal activity within the basal ganglia, i.e., CPu, GP, and STN are abnormally modified after acute MPTP treatment although further electrophysiological experiments are necessary to demonstrate more clearly the effect of MPTP on neuronal activities. Taken together, we found that acute MPTP treatment can induce astroglial and microglial activation in STN and result in neuronal dysfunction of basal ganglia in parallel to the degeneration of SNpc dopaminergic neurons. Finally, we examined whether or not acute MPTP treatment induces astrocytic and microglial activation, and degeneration of dopaminergic neurons (Fig. 5), or neuronal activity changes (Fig. 6) in the spinal cord. Acute MPTP treatment changed the number or shape of neither astrocytes (Fig. 5a, b) nor microglia (Fig. 5c, d) in the lumbar spinal cord compared to saline treatment. In addition, there was no difference in TH-immunopositive neurons (Fig. 5e, f), MAP2 immunoreactivities (Fig. 6a, b), and the expression of c-Fos in both spinal dorsal and ventral horns (Fig. 6c, d, e) after acute MPTP treatment suggesting that there is no degeneration of neurons including dopaminergic neurons in acute MPTP model. Therefore, these results suggest a central mechanism of painful sensation related to PD and provide a basis for substantial beneficial effects of STN deep brain stimulation (DBS) (STN-DBS) in PD patients [10, 11].

## Discussion

In this study, we showed that depletion of dopaminergic neurons in the nigrostriatal pathway by acute intraperitoneal injection of MPTP induced an alteration of behavioral responses to mechanical and thermal stimulation. In the rotarod test, which is widely used to confirm motor deficits in rodent PD models [21], MPTP-treated mice exhibited clear motor deficits. However, the distance moved and basal anxiety level in the OFT was not affected by MPTP administration, which was consistent with other studies [14, 22]. To exclude explorative behavior in the open field, we additionally performed the round chamber test and confirmed that mice displayed locomotor dysfunction after MPTP injection.

MPTP-treated mice exhibited more sensitive nociceptive behavior when mechanical stimulation was introduced. MPTP-treated mice showed shorter paw withdrawal latencies in the automated von Frey test compared to saline-treated counterparts (Fig. 3a). This indicated that hypersensitivity to mechanical stimuli was elicited by MPTP injection. A similar effect was observed with the administration of 6-OHDA in rats, which increased the nociceptive response to mechanical stimulation compared to controls [5]. MPTP-treated mice also exhibited a lower nociceptive threshold for thermal stimuli compared to saline-treated mice, suggesting modified behavioral response to thermal stimulations by intraperitoneal MPTP administration (Fig. 3b, c). Although we did not investigate alterations in the response latency to thermal stimuli during the PD induction phase in this mouse, our results revealed the effects of MPTP on thermal nociceptive thresholds might show up after PD induction, which was consistent with a previous study describing the long-term effects of MPTP [21].

In PD, the cause of pain and pain relief management remains unknown and there is no existing therapeutic tool for pain. In the present study, we found that the degenerative loss of dopaminergic neurons in SNpc induced astrogliosis and microglial activation related to inflammation and neuronal dysfunction in the basal ganglia including CPu, GP, and STN, which has a dopaminergic connection from the SNpc, but not in the spinal dorsal horn (known to be a key site for pain processing). In other studies, 6-OHDA injection into the striatum generated a moderate increase in astrocyte activation in the GP [17], and an MPTP-treated chronic PD mouse model showed a loss of motor neurons and activation of astrocytes in the spinal cord [23]. The discrepancy between these results and our findings is likely due to the protocol used to induce the PD model: (1) MPTP vs. 6-OHDA, and (2) MPTP-chronic vs. MPTP-acute models. Although the mechanisms underlying remote local activation of astrocytes and microglia after MPTP injection remains uncertain, the local inflammatory responses in the STN in response to SNpc dopaminergic neuronal loss might be the key point to be discussed in the view of pain mechanisms and pain therapeutics such as deep brain stimulation (DBS) of STN in PD. Indeed, recent clinical data demonstrated the therapeutic effect of STN-DBS on pain in PD patients [10, 11], although the exact mechanism of relief is still a matter of debate. In addition, it is already well known that abnormal overactivity of GP neurons by glutamatergic input from STN involves in the motor symptoms of PD and STN-DBS can control the overactivity of STN-GP pathways leading to slow progression of motor symptoms in PD [24].

The basal ganglia have roles not only in attention, cognition, and stress [25–27], but also in movement and pain, as well-indicated in PD patients [28, 29]. Besides the GP and STN, the striatum comprised of caudate-putamen is the largest of four major nuclei in the basal ganglia. The dopaminergic system in the striatum has been implicated in pain. Inhibition or activation of striatal dopamine D2 receptors is related to nociceptive responses [4]. Human studies also support the function of dopamine D2 receptors in pain [30]. In line with the studies mentioned above, here we found that acute MPTP administration significantly reduced dopaminergic fibers and highly increased the neuronal activity in the striatum, especially in dorsal striatum. Moreover, MPTP-induced dopaminergic neuronal degeneration lowered nociceptive thresholds in mice. These results suggest that the dopaminergic nigrostriatal pathway also has a role in inhibiting basal mechanical and thermal nociception. However, the nigrostriatal pathway or SNpc-STN neuronal circuitry may not be the only pathway to modulate nociceptive thresholds to mechanical and thermal stimuli leading to pain suppression. The dopaminergic system in the spinal cord also influences motor function as well as nociception, and dopamine D2 and D3 receptors in the spinal cord play a role in anti-nociception [31] leading to descending inhibition [32].

On the other hand, although it has been reported that MPTP induces lesions selectively of dopaminergic neurons [33], intraperitoneal administration of MPTP can induce the depletion of dopaminergic neurons in the forebrain as well as spinal cord [34] affecting descending facilitation of pain. Increased expression of D2 receptor in anterior cingulate cortex is correlated with nociceptive pain in neuropathic pain [35] and inflammatory nociception results in the increase of D2 receptors in the insular cortex [36]. In addition, inflammatory responses or dopamine D1 receptor of spinal cord can mediate pro-nociceptive actions [37]. In our study, however, we did not find any changes in astroglial/microglial activation, the number of TH-immunopositive neurons, and neuronal activity after acute MPTP treatment. Taken together, we suggest that pain symptoms of PD induced by acute MPTP administration could be related to supraspinal mechanism in the modulation of pain rather than spinal mechanism. Thus, apart from inflammatory activation and neuronal activity using c-Fos expression, further studies about alterations of basal nociceptive thresholds induced by depletion of dopaminergic neurons at the spinal cord and cortical level are clearly needed.

In summary, the present results confirm that intraperitoneal acute MPTP administration in mice induces degeneration of dopaminergic neurons and fibers in the striatum and the SNpc, increased neuronal activity in the striatum and GP, and inflammatory activity in the STN, which are all involved in nociception. Although motor deficits were detected in MPTP-treated group, their exploration level in the OFT was normal and even their pain behavioral response latencies to mechanical and thermal stimuli were significantly shorter than the saline-treated group. Taken together, MPTP-induced mouse PD model can be used to investigate pain symptoms and central pain processing mechanisms related to PD.

## Conclusions

The abnormal nociceptive threshold and pain perception which is the most common non-motor symptoms could be related to dysfunctional neural circuitry induced by inflammatory responses such as astrogliosis and neural activity within basal ganglia including striatum, globus pallidus, subthalamic nucleus, and substantia nigra in acute Parkinson’s disease.

## Materials and methods

### Animals and drug administration

Experiments were performed with 8–9 weeks old male C57BL/6J mice (19 ~ 24 g) because female mice have much higher sensitivity than male mice to noxious stimuli due to the biological factors including sex hormones’ effect [38]. Mice were group housed and kept under conditions of a 12 h light/dark cycle with an *ad libitum* food and water supply. All experiments were approved by Seoul National University or Kyungpook National University Institutional Animal Care and Use Committees.

#### MPTP treatment

Mice were injected intraperitoneally (i.p.) with MPTP (20 mg/kg, Sigma, M0896) or sterile saline solution (four times at 2 h intervals) [3].

#### L-DOPA treatment

L-DOPA (Sigma, D1507) was dissolved in sterile saline. Mice were injected subcutaneously with L-DOPA (40 mg/kg) or sterile saline solution. All tests were performed at 45 min post-injection [39].

### Behavioral tests

Mice were held in their home cage for 30 min before the behavioral tests.

#### Rotarod

The rotarod training was performed at the same time for 10 min during five consecutive days: on the first day, mice were placed on the rotating rod at 4 rpm for 5 min. Every 30 s, the rod speed was increased by 1 rpm up to 15 rpm, and then maintained this speed for one minute. On the second day, a 4 rpm speed was used for 1.5 min. The rod speed was increase by 1 rpm every 30 s until it reached 20 rpm, which was maintained for 10 min. On the third, fourth, and fifth days, the latencies of falling off rod were measured three times with linear increase of rod speed from 4 rpm up to 40 rpm for 5 min and averaged. The actual test was performed after the drug treatment. The test protocol was the same with the last three days of the training protocol.

#### Open field test (OFT)

Each mouse was placed in an open field arena (40 cm x 40 cm x 40 cm) made of white acrylic and video monitored from above for 5 min. Total distance moved and total time spent in three zones (10 cm x 10 cm, 20 cm x 20 cm for center and 40 cm x 40 cm excluding the center area for the peripheral zone) were calculated using ETHOVISION 9.0 software (Noldus).

#### Round chamber test

The mice were placed in a round chamber (15 cm diameter, 10 cm height) for 10 min and video-recorded from above. The video recording files were analyzed using ETHOVISION 9.0 software (Noldus) [40, 41].

#### Dynamic plantar aesthesiometer

To measure the mechanical nociceptive threshold, mice were habituated in a Dynamic Plantar Aesthesiometer (Ugo Basil, 37,450) for 30 min (or more) until they stabilized. Ascending force was given to the hind paw of the mice until paw withdrawal occurred. The force increased from 0 to 5 g over a 10 s period (0.5 g/s), and 5 g force was maintained for an additional 10 s [42].

#### Hot plate test

The thermal nociceptive responses were measured by placing mice on a hot plate (Harvard Apparatus) having a constant temperature (48 °C or 53 °C). The latency of flinching, licking, or jumping behavior was recorded. In order to prevent tissue damage at 53 °C, a cut-off time of 20 s was employed [43].

#### Tail-flick test

The thermal nociceptive responses of the tail were measured by tail-flick apparatus (LE7106 Tail-flick Meter). Laser stimulation was applied to the dorsal surface of the tail until the tail was flicked. The beam light intensity was adjusted to have 4–6 s of tail flick latencies for the average baseline. The latency time was measured three times with 3 min intervals. The light was focused on different points of the tail (1 cm apart) in each trial.

### Immunohistochemistry

Mice were anesthetized by ketamine injection and transcardially perfused with 25 ml of phosphate buffered saline (PBS), followed by 25 ml of 4 % paraformaldehyde (PFA) dissolve in PBS. Brains were removed and kept in 5 ml of 4 % PFA in PBS overnight at 4 °C, and then submerged for 48 h at 4 °C in 30 % sucrose dissolved in PBS. Brains were frozen in frozen section compound (Leica #3801480) and serial coronal sections were made on a cryostat at 30 μm thickness. Twenty-four brain slices including caudate-putamen and globus pallidus (GP), forty-two slices of SNpc, and twelve slices of subthalamic nucleus (STN) were obtained and conserved in 50 % glycerol (in PBS). Free-floating four striatum/GP brain slices, seven SNpc slices, and two STN slices from each mouse were rinsed three times for 5 min in PBS at room temperature (RT) and incubated in blocking solution (2 % goat serum, 1 % bovine serum albumin (BSA) and 0.5 % Triton X-100, in PBS) for 1 h. The sections were incubated with primary antibodies against tyrosine hydroxylase (TH) (1:1000, abcam, ab76442), glial fibrillary acidic protein (GFAP) (1:1000, Dako, USA), Iba1 (1:1000, abcam, ab5076), NeuN (1:1000, Millipore, USA), and c-Fos (1:1000, Santa Cruz, SC-52, USA) in blocking solution overnight at 4 °C, and then secondary antibodies, anti-goat or anti-rabbit Alexa Fluor 488 or 594 IgG (1:300, Invitrogen) for 2 h at RT. The relative intensity level of TH-immunoreactive fibers in the striatum was measured using ImageJ program. The TH-immunoreactive neurons in the SNpc were counted by ImageJ program with Point Picker plugin.

For immunohistochemistry of spinal neurons, astrocytes, and microglia, the same procedures mentioned above were applied. Mice were anesthetized by ketamine at 30 min after the last injection of MPTP. Lumbar and sacral spinal cords were dissected out after transcardinal perfusion with fixatives and 48 spinal cord slices were obtained. Spinal neurons and astrocytes in six lumbar-sacral slices from each mouse were labeled with primary antibodies against microtubule-associated protein 2 (MAP2), GFAP, Iba1, and c-Fos, respectively. The c-Fos expression analysis was done as described in Yu et al. [44]. The relative intensity of immunopositive cells for MAP2, GFAP, Iba1, and c-Fos in the spinal cord was measured using ImageJ program. The immunoreactive neurons and astrocytes were counted by ImageJ program with Point Picker from four among the six slices.

### Western blotting

Coronal brain slices (400 μm thickness) including striatum or SNpc were prepared with a vibratome (Intracel Ltd), and homogenized in RIPA buffer comprising (in mM): TriCl 50 at pH 7.6; NaCl 150; EDTA 1; DTT 1; NP-40 1 %; SDS 0.1 %; sodium deoxycholate 0.5 %. Immunoblotting was performed using tyrosine hydroxylase (TH) (1:1000; Millipore), actin (1:10,000; Invitrogen) and then goat anti-rabbit IgG (1:5000; Cell Signaling). Bands on nitrocellulose (NC) membrane were visualized using a Chemiluminescent HRP Substrate kit (Millipore) and ChemiDoc XRS+ (721BR02655, Bio-Rad). ImageJ program was used to quantify the band intensity.

### Data analysis

All data were plotted on graphs and statistical analyzed using the GraphPad Prism 5.01 program.

## Abbreviations

D: Dopamine receptor
DBS: Deep brain stimulation
GFAP: Glial fibrillary acidic protein
GP: Globus pallidus
L-DOPA: L-3,4-dihydroxyphenylalanine
MAP2: Microtubule-associated protein 2
MPTP: 1-methyl-4-phenyl-1,2,3,6-tetrahydropyridine
OFT: Open field test
6-OHDA: 6-hydroxydopamine
PD: Parkinson’s disease
SNpc: Substantia nigra pars compacta
STN: Subthalamic nucleus
TH: Tyrosine hydroxylase
